# Comparison, alignment, and synchronization of cell line information between CLO and EFO

**DOI:** 10.1186/s12859-017-1979-z

**Published:** 2017-12-21

**Authors:** Edison Ong, Sirarat Sarntivijai, Simon Jupp, Helen Parkinson, Yongqun He

**Affiliations:** 1 Department of Computational Medicine and Bioinformatics, University of Michigan, Ann Arbor, MI USA; 2 Samples, Phenotypes, and Ontologies Team, European Bioinformatics Institute (EMBL-EBI), European Molecular Biology Laboratory, Hinxton, Cambridge, UK; 3 Center of Computational Medicine and Bioinformatics, University of Michigan, Ann Arbor, MI USA; 4 Unit of Laboratory Animal Medicine, University of Michigan, Ann Arbor, MI USA

**Keywords:** Cell line, Experimental factor ontology, Cell line ontology, Data integration, Data mapping

## Abstract

**Background:**

The Experimental Factor Ontology (EFO) is an application ontology driven by experimental variables including cell lines to organize and describe the diverse experimental variables and data resided in the EMBL-EBI resources. The Cell Line Ontology (CLO) is an OBO community-based ontology that contains information of immortalized cell lines and relevant experimental components. EFO integrates and extends ontologies from the bio-ontology community to drive a number of practical applications. It is desirable that the community shares design patterns and therefore that EFO reuses the cell line representation from the Cell Line Ontology (CLO). There are, however, challenges to be addressed when developing a common ontology design pattern for representing cell lines in both EFO and CLO.

**Results:**

In this study, we developed a strategy to compare and map cell line terms between EFO and CLO. We examined Cellosaurus resources for EFO-CLO cross-references. Text labels of cell lines from both ontologies were verified by biological information axiomatized in each source. The study resulted in the identification 873 EFO-CLO aligned and 344 EFO unique immortalized permanent cell lines. All of these cell lines were updated to CLO and the cell line related information was merged. A design pattern that integrates EFO and CLO was also developed.

**Conclusion:**

Our study compared, aligned, and synchronized the cell line information between CLO and EFO. The final updated CLO will be examined as the candidate ontology to import and replace eligible EFO cell line classes thereby supporting the interoperability in the bio-ontology domain. Our mapping pipeline illustrates the use of ontology in aiding biological data standardization and integration through the biological and semantics content of cell lines.

**Electronic supplementary material:**

The online version of this article (10.1186/s12859-017-1979-z) contains supplementary material, which is available to authorized users.

## Background

Tens of thousands of cell lines have been developed and used in experimental research, making the usage of cell lines a major tool for scientific discovery. A biomedical ontology is a set of human- and computer-interpretable terms and relations that represents various entities and the relations among these entities in a biomedical domain. Biomedical ontologies are critical to data and knowledge representation, standardization, integration, and computer-assisted reasoning. To better support cell line-based research, it is critical to have a standardized ontology that represents available cell lines, their associated cell types, tissues, and diseases, and how these entities connect to each other.

The Experimental Factor Ontology (EFO) is a data-driven biomedical application ontology developed by the European Bioinformatics Institute (EMBL-EBI) to organize the diverse experimental variables needed to describe data resided in the EMBL-EBI resources, including cell line data [1]. EFO aims to build on and extend existing reference ontologies from the OBO foundry, such as the Human Phenotype Ontology [2] and the Uberon anatomy ontology [3], and uses a number of design patterns to integrate and cross-link these ontologies. EFO includes many cell lines that were created to annotate cell lines from experiments in the Array Express archive [4]. Aligning the EFO cell lines with the community-based Cell Line Ontology (CLO), which describes immortalized cell lines [5], would enable semantic alignment of cell lines and associated datasets. There also exist other resources that attempt to bridge cell line information from EFO and CLO together, such as Cellosaurus [6]. Cellosaurus is a manually curated knowledgebase of cell line resources for biomedical research, and it provides high quality cross references to other resources including EFO and CLO, which can be used to facilitate cross-linking between the two resources.

Currently, EFO cell line is loosely defined as a population of cell units and covers primary cell lines as well as permanent cell lines under ‘material entity’. On the other hand, CLO defines characteristics of cell line at a single-cell level and aims to cover the immortalized stable cell lines. With the different conceptual viewpoint of EFO and CLO cell lines, the population level definition of EFO cell line is suitable to model experiments where measurement is performed on cell line cultures. Even though cell line cultures were assumed homogeneous, recent studies reviewed the effects of heterogeneity of cells within the same culture [7–9]. The analysis of single-cell dynamics in the culture can increase our understanding of cellular level interaction and provides a better assessment of cell behavior in the culture. Thus, CLO’s cell line definition as individual cells complements data modeling of single-cell experiment. The different aspects of cell lines in CLO and EFO (individual cell versus population of cells) are complementary components of each other to describe a cell line related information, knowledge integration and collaboration. The complementary modeling of both approaches has been discussed in the CLO paper [5].

Besides the difference in the definition of individual-level and population-level of cell lines, EFO and CLO also have different coverages. EFO covers primary cell lines, stem cell derived cell lines, and immortalized permanent cell lines that can be cross-referenced with central cell line catalogs, while CLO only covers immortalized permanent cell lines. While the aim of this study is to align cell lines in CLO and EFO, cell lines that do not belong to the immortalized permanent cell lines mapping described above should remain in the scope of EFO-native primary cell lines.

In this study, we have developed a mapping process that combines third-party cross referencing, lexical and biological content comparison, and semantic relation matching to compare, align and synchronize immortalized permanent cell lines available in EFO and CLO. This will allow for a better cell line knowledge integration within the scope of the OBO Foundry [10]. Additional cell line information obtained from EFO, such as organs and diseases, will be incorporated into CLO using the design pattern developed in this study. Cellosaurus was used as a high-quality third-party cell line resource to verify mapping between EFO and CLO, as well as the identification of immortalized permanent cell lines in EFO to be included in CLO. The information of EFO-CLO aligned cell line will be merged from the two ontologies. Mapped EFO cell line classes will be deprecated and replaced by the corresponding CLO cell line classes with CLO namespace. Additional immortalized permanent cell lines from EFO will be added to CLO and assigned with new CLO URIs. This work resulted in an updated CLO ontology with EFO-CLO aligned information which will be used as the source ontology for cell lines in future EFO’s production.

## Methods

### Data preparation

**Fig. 1:**
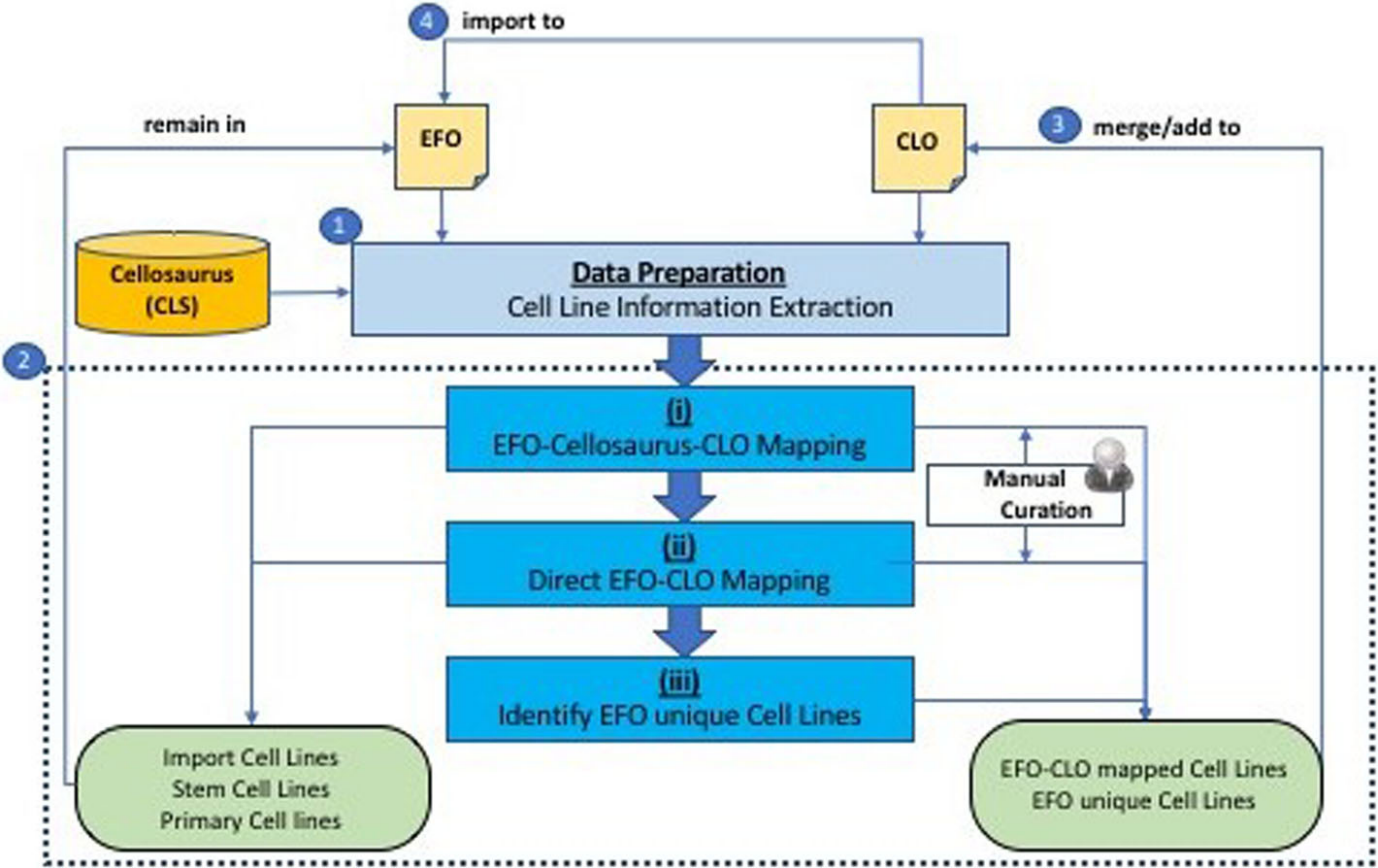
General project workflow. The general pipeline of comparing, aligning and synchronizing cell lines between EFO (version 2.85) and CLO (version 2.1.106) with additional information downloaded from Cellosaurus (version 22.0).The workflow was separated into four major steps (indicated as *blue circles*). Step 1: Cell lines and related biological information were downloaded and extracted from EFO, CLO and Cellosaurus. Step 2: Comparison and alignment of EFO cell lines to CLO through three intermediate processes: (i) EFO-Cellosaurus-CLO Mapping that performed cross-references and validations among the three cell line resources; (ii) Direct EFO-CLO Mapping that compared and mapped EFO cell lines to all CLO cell lines; (iii) Identification of EFO unique Cell Lines that were immortalized permanent cell lines not available in CLO. The results of (i)-(iii) were summarized in Fig. 4.EFO cell lines with foreign (non-EFO) namespace such as Brenda Tissue Ontology (BTO), stem cell lines and primary cell lines were excluded from the mapping and remained in EFO. The overall mapping result was summarized in Table 1. Step 3: The mapped EFO-CLO cell lines and EFO unique immortalized permanent cell lines would be merged or added to CLO. Step 4: The updated and synchronized CLO will later be imported to EFO immortalized permanent cell line module

**Fig. 2:**
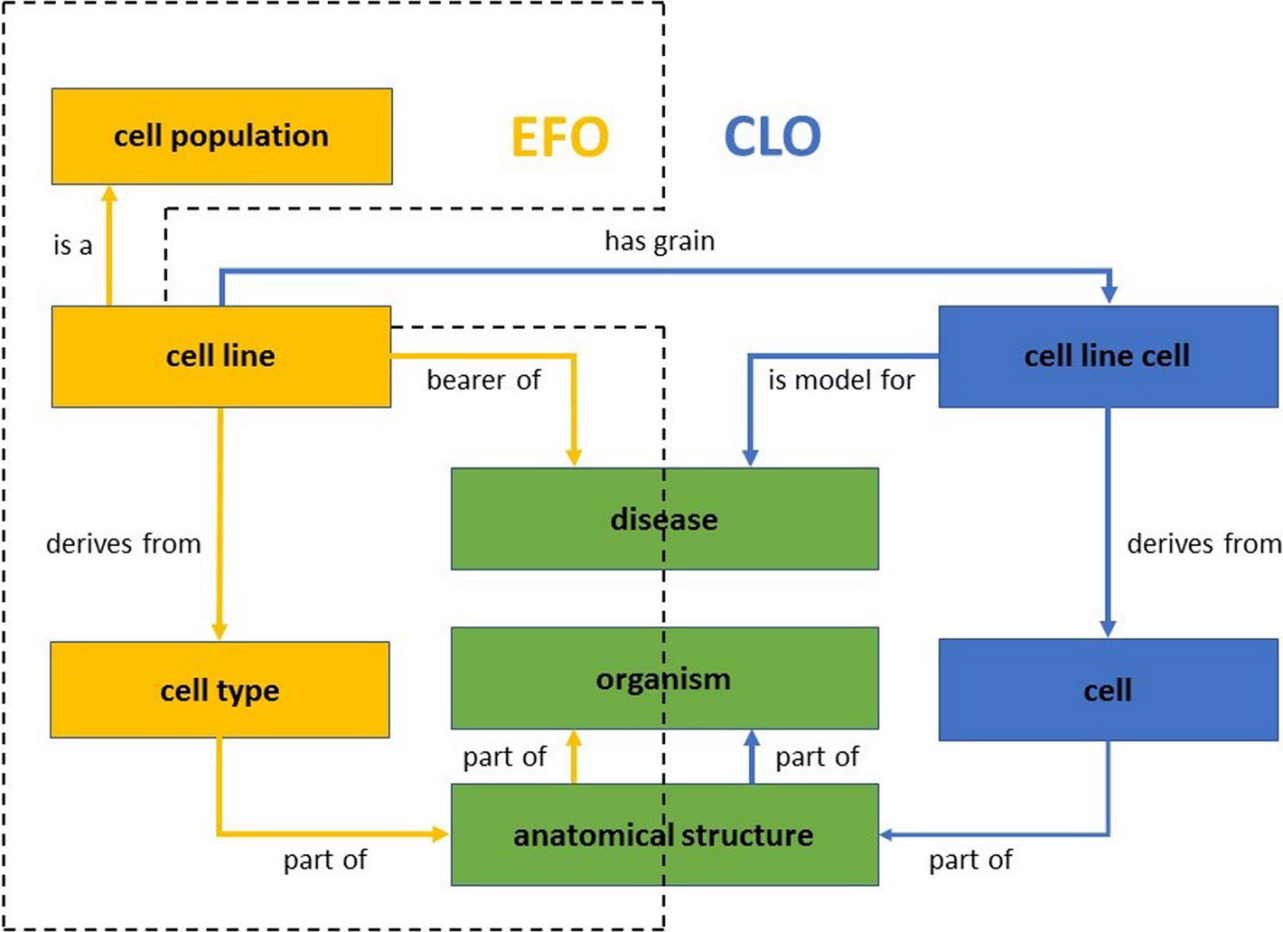
Comparison of EFO and CLO cell line design patterns. The EFO cell line design pattern was colored in *orange* and CLO in *blue*. The *green* color indicated cell line related information and design pattern shared in both EFO and CLO design patterns

The input ontology OWL files of EFO (version 2.85) and CLO (version 2.1.106) were downloaded from Ontobee [11] and the text format of Cellosaurus (version 22.0) was downloaded from ExPASy portal [12]. Additionally, EFO cell lines that were drawn from external sources (e.g., CLO and BRENDA Tissue and Enzyme Source Ontology (BTO) [13]) were excluded (Additional file 1: Table S1) from the mapping process as the aim of this study is to map EFO-namespaced cell lines to referenced CLO cell lines. Cell lines and its annotations (label, synonyms and cross references) under EFO’s ‘cell line’ class, CLO’s ‘cell line cell’ class and all Cellosaurus cell lines, along with the related biological information (disease, cell type, anatomical location and species of origin), were extracted as depicted in Fig. 1 Step 1 (blue circle). Specifically, for EFO and CLO, the cell line related information was stored as semantic axioms with different design patterns as illustrated in Fig. 2. The rationale of examining both lexical contents and biological information is to ensure the mapping accuracy between CLO and EFO cell lines. Two cell lines of an identical or very similar label do not always represent the same cell line. For example, Cell line 17/14 (accessioned HB-8153 in ATCC catalog) is not the same cell line as 171-4 (accessioned HB-296 in ATCC catalog). Normalizing these two cell lines by removing punctuation marks will result in a false normalization [14]. After extracting annotations and related information from the three resources, the mapping pipeline was divided into three consecutive steps.

### EFO-Cellosaurus-CLO mapping

**Fig. 3:**
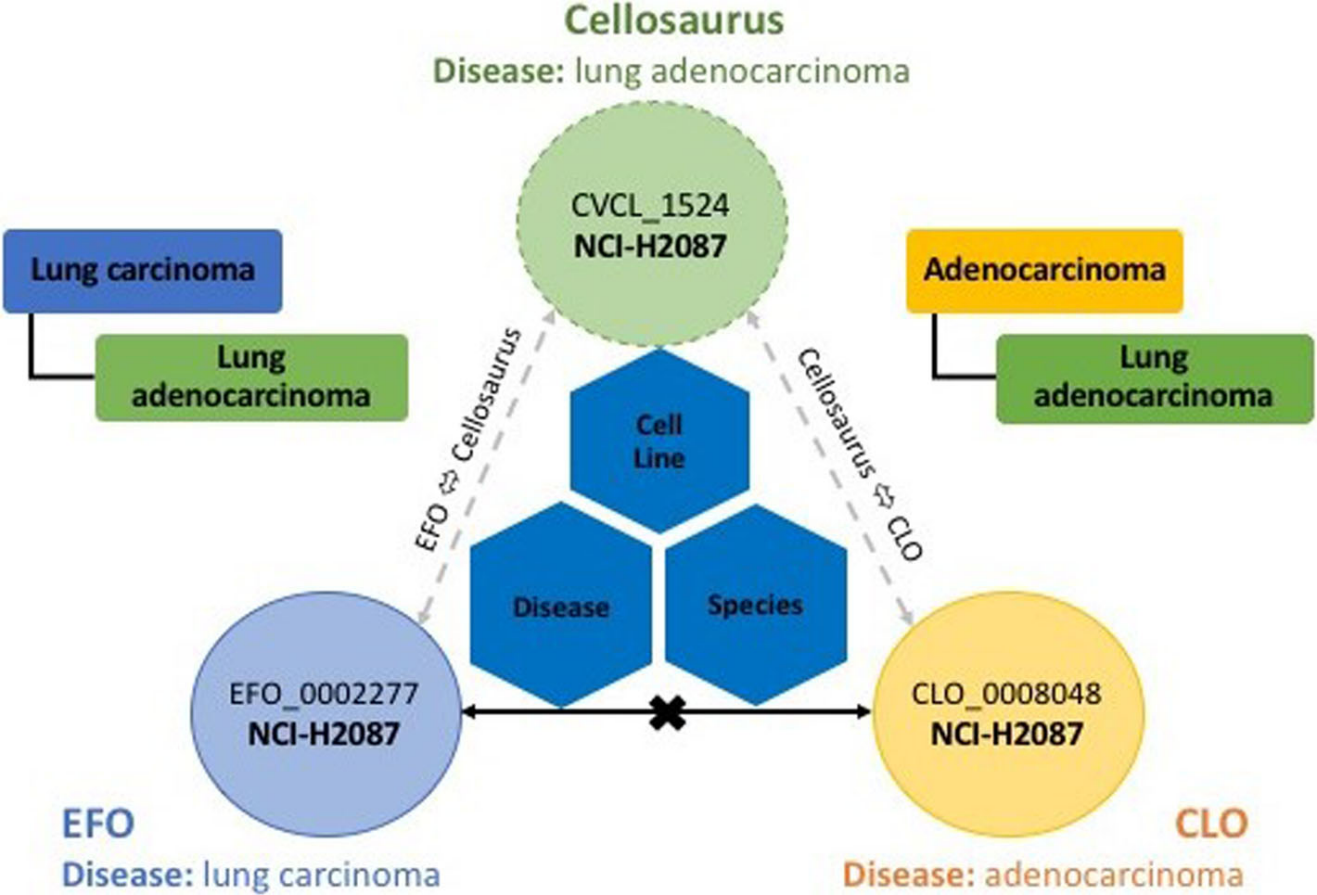
Example EFO-CLO cell line mapping recovered by Cellosaurus disease definition and semantics matching. There were three different disease definitions (“lung carcinoma” in EFO, “lung adenocarcinoma” in Cellosaurus and “adenocarcinoma” in CLO) for the cell line “NCI-H2087”. The direct mapping using cell line annotations, disease and species of origin would have gone undetected if we directly compared EFO cell line disease information to CLO’s information. Discrepancies of cell line-disease annotation can be recovered through EFO-Cellosaurus-CLO disease semantic relations

In order to achieve cell line mapping with high confidence and quality, a three-way mapping among EFO, CLO and Cellosaurus was first performed (Step 2, process (i) in Fig. 1). Only EFO and CLO cell lines with unique cross reference to Cellosaurus were aligned in this step, and EFO cell lines with multiple non-unique cross references to Cellosaurus were directly matched against CLO in the following step for validation. Due to limited cell line information available in Cellosaurus, only cell line annotation property values (name, synonyms, and cross reference) and the common information shared in both EFO and CLO (disease and species of origin) were checked to validate the mapping. Furthermore, if the diseases, each defined for a cell line from each resource, had a direct subclass-superclass relationship, these two diseases would be considered as matched. For example, the cell line “NCI-H2087” had three different disease definitions, “lung carcinoma”, “lung adenocarcinoma” and “adenocarcinoma” in EFO, Cellosaurus and CLO, respectively (Fig. 3). The direct matching of this cell line between EFO and CLO would not be valid because of the poorly defined disease association, but such mapping could be recovered by the direct subclass-superclass relation of diseases in EFO-Cellosaurus (“lung carcinoma” to “lung adenocarcinoma”) and CLO-Cellosaurus (“adenocarcinoma” to “lung adenocarcinoma”). Cell lines that had unmatched cell line annotations or cell line related information were manually verified.

### Direct EFO-CLO mapping

EFO cell lines that were not processed in the previous step were directly mapped to CLO cell lines (Step 2, process (ii) in Fig. 1). A confidence score (C-score) was developed to score the confidence of mapping between an EFO cell line and all CLO cell lines.

$$ Confidence Score=\sum \limits_{i=1}^KM- SLD $$

$$ where\ M=\left\{\begin{array}{c}\kern0.5em +1\\ {}-1\\ {}0\end{array}\right.\kern0.75em {\displaystyle \begin{array}{c} matched\\ {} unmatched\ \\ {} missing\end{array}}{i}^{th}\  cell line related element $$

$$ K\subseteq \left\{ disease, cell type, anatomic location, species of origin\right\} $$

*SLD* is shortest Levenshtein distance (*SLD*) among all the combinations of EFO and CLO cell line labels and synonyms [15]. In brief, the Levenshtein distances were computed for every pair of cell line names (label or synonyms) from EFO and CLO, and the smallest Levenshtein distance was selected as the SLD. In the case of an exact label and synonyms match or an exact cross reference match of the EFO and CLO cell line, the value of SLD is zero. *M* is a function indicating whether the *i* ^*th*^ cell line related element in *K* was matched (+1) between EFO and CLO cell line, or unmatched (−1). *K* contained the cell line related information including diseases, cell type, anatomic location and species of origin extracted from the corresponding ontologies. The C-scores of each EFO cell line against all CLO cell lines were computed. For example, the mapping between EFO cell line (EFO_0002208) NIH3T3 and CLO cell line NIH-3 T3 (CLO_0004301) had *SLD* of 1 (insertion of a special character, “-”) and ${\sum}_{i=1}^KM$ of +3 (matched disease, anatomic location and species of origin; missing cell type information), which gave a C-score of +2. Since it is too labor intensive to go through thousands of EFO-CLO matches for one EFO cell line, only top three CLO cell lines with the best C-scores mapped to an EFO cell line were inspected. Based on the calculated C-score, the mapping could be summarized into three categories: exact cell line annotation match with valid cell line related information match; exact cell line annotation match with invalid cell line related information match that required manual validation; inexact cell line annotation match that required manual selection from the top three mapped CLO cell lines. The remaining unmapped EFO cell lines after manual selection then go through the last step of the mapping pipeline.

### Identification of additional EFO immortalized permanent cell lines

Since there are biological and modeling differences between primary cell lines and immortalized permanent cell lines, it is necessary to check whether the remaining unmapped cell lines from the previous step were in fact immortalized permanent cell lines or not. There is not an explicit statement that distinguishes immortalized cell lines from primary cell lines in the EFO cell line classes. The identification of additional immortalized permanent cell lines from EFO was done by cross referencing to Cellosaurus since primary cell lines are not curated in the Cellosaurus (Step 2, process (iii) in Fig. 1). Therefore, if there is a traceable record of the cell line in Cellosaurus, we made an assumption of it being a permanent immortalized cell line and should be added to CLO. EFO cell lines with cross reference in the Cellosaurus that are not listed in the cell type category of “stem cell” were considered as “immortalized permanent cell line” and would be added to CLO, relying on the high-quality cell line annotations manually curated in the Cellosaurus for verification. Cellosaurus is a comprehensive collection of permanent cell lines and does not contain hierarchical classification, we could not assume a subClassOf relationship for these cell lines. For EFO cell lines that were under the “stem cell” category or not cross-referenced in Cellosaurus would be kept in the EFO namespace because CLO does not yet support stem cell derived cell lines. Additionally, the EFO cell lines without Cellosaurus cross references were manually verified to be primary cell lines. The scope CLO is to cover immortalized permanent cell lines deposited in major cell line repositories such as ATCC, Coriell, HyperCLDB and Riken, and does not have the design patterns for primary cell lines nor stem cell and stem cell derived cell lines. Therefore, these EFO primary cell lines, as well as the stem cell and stem cell derived cell lines were not mapped to CLO.

### Programming implementation

Alignment by cross-referencing between CLO and EFO was mined from Cellosaurus knowledgebase. The source files of EFO and CLO were then processed for ontology class property alignment. A set of regular expression rules were designed for normalization of special characters and comparison of cell line rdfs:label property. The semantics and other biological information enriched by CLO’s and EFO’s design patterns were retrieved and compared using the OWL API library [16]. The changes reflecting the EFO-CLO alignment will be updated in CLO and deposited onto CLO GitHub repository where EFO can further subsume and import into EFO replacing locally identified terms. Permanent URLs (PURL) of both ontologies are also resolved on the EMBL-EBI Ontology Lookup Service (OLS) [17], the EMBL-EBI RDF Platform [18] and the Ontobee [11]. All information can be queried using SPARQL in Ontobee SPARQL endpoint (http://www.ontobee.org/sparql).

### Building cell line design pattern

Cell lines in CLO and EFO have different naming strategies. In EFO, cell line naming convention is a mix of individual cell implication (e.g., HEK-293 cell), or a cell line name with no indication of cell population (e.g., MCF 10A). CLO focuses on the definition of individual ‘cell line cell’ and its laboratory-derived descendants. CLO:‘cell line’ is a population of CLO:‘cell line cells’. The definition of ‘cell line cell’ targets individual single cells, which offers the advantage of using CLO’s design pattern in single cell profiling data representation. Therefore, the aforementioned EFO’s cell line examples would be converted to ‘HEK-293 cell’ and ‘MCF 10A cell’ according to CLO’s naming convention to aid normalization between the two resources. The normalization also improves the readability and clarity of EFO cell line names when working in OBO space.

The design patterns of both EFO and CLO were similar with some minor variation, and CLO design pattern was used as the template to bridge EFO unique features (Fig. 2). First, a cell line in CLO was defined as a biologically individual cell (“cell line cell”) while in EFO cell line was described as cell populations (“cell line”), and the two classes were linked by object property “*has grain*” in CLO. Even though cell line related biological information was mostly shared between EFO and CLO, two minor differences existed. First, EFO uses the object property “*bearer of*” to model disease while CLO uses “*is model for*”. Second, CLO “cell line cell” was “*derives from*” the CL class “cell” which can be “cell type” or another “cell line cell” (which is also a cell type), but in EFO “cell line” was “*derives from*” only “cell type”. Therefore, aligning EFO with CLO by importing CLO classes into EFO required adjustment within EFO so the differences in the two cell line design patterns were resolved.

## Results

**Fig. 4:**
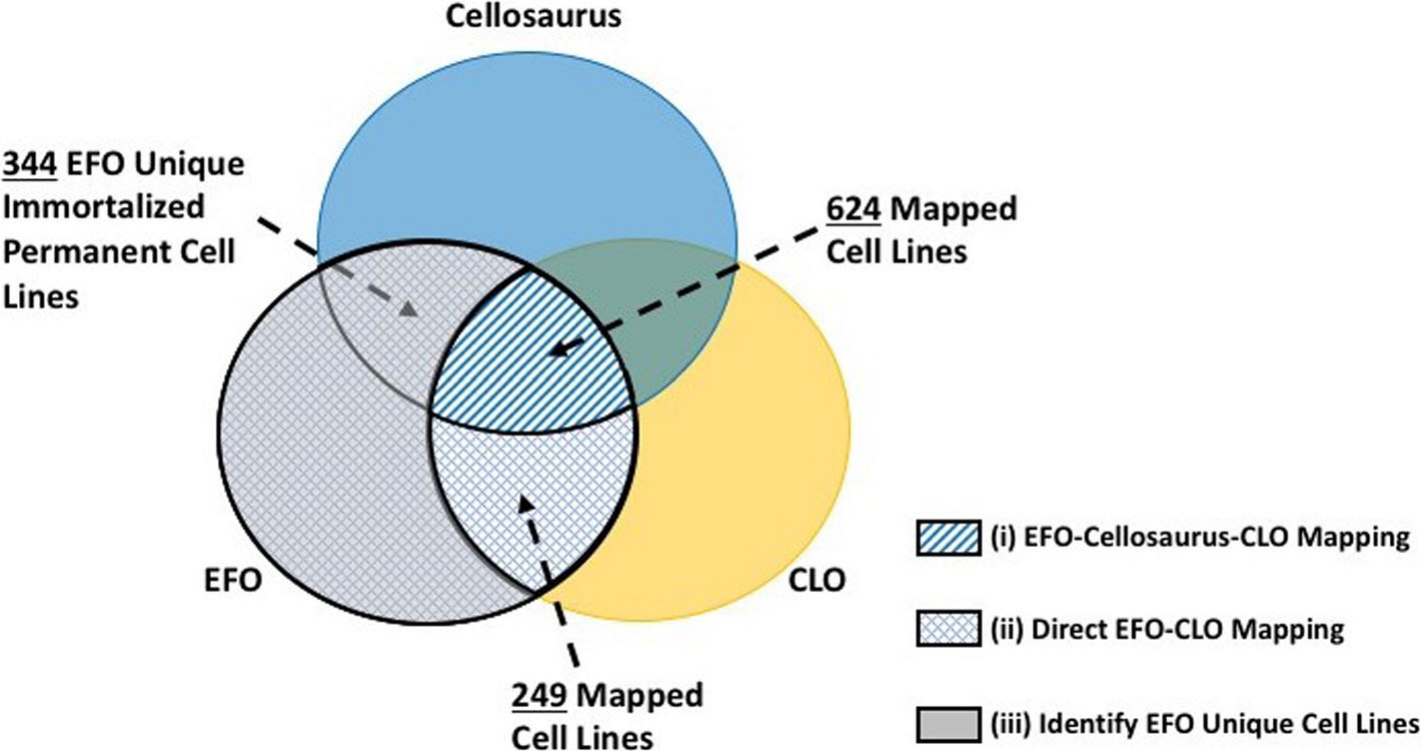
Mapping results of the three intermediate processes (i)-(iii) in Step 2 of the overall workflow. (i) EFO-Cellosaurus-CLO Mapping shown with diamond pattern. (ii) Direct EFO-CLO Mapping was the region with diamond pattern. In this intermediate process. (iii) Identify EFO Unique Cell lines was the shaded region overlapped with (ii). All the unmatched EFO cell lines from (ii) were processed in (iii) to identify EFO unique immortalized permeant cell lines that would be added to CLO

The results of the three intermediate steps, (i) EFO-Cellosaurus-CLO mapping, (ii) Direct EFO-CLO Mapping and (iii) Identify EFO Unique Cell Lines were illustrated in Fig. 4. The overall mapping result was summarized in Table 1. In conclusion, there were 874 EFO-CLO mapped cell lines aligned and cell line related information would be merged into CLO and 344 EFO unique immortalized permanent cell lines added to CLO (Additional file 2: Table S2). These merged or added EFO-CLO cell lines could be imported from CLO as the immortalized permanent cell line module into EFO with enriched cell line information integrated from EFO and CLO. Since CLO does not cover primary cell lines nor stem cell and stem cell derived cell lines, the 66 stem cell lines and 32 primary cell lines identified at the end of this study would remain in the EFO namespace until further investigation.

**Table 1:** EFO-CLO cell line mapping results. The EFO-CLO mapping result from Step 2 (dotted frame) of Fig. 1. The EFO-CLO mapped cell lines and EFO unique immortalized permanent cell lines were to be merged or added into CLO. The imported cell lines (EFO cell lines with foreign CLO or BTO namespace), stem cell lines and primary cell lines would be kept in EFO core ontology

Type of Mapping Result	# of Cell Line
EFO-CLO Mapped Cell Lines	873
EFO Unique Immortalized Permanent Cell Lines	344
Imported Cell Lines	448
Stem Cell Lines	66
Unmapped Cell Lines	32

### Improved cell line mapping by biological and semantic content

Using the 624 cell lines in the EFO-Cellosaurus-CLO mapping step as the gold standard, we compared and evaluated the improvement of cell line mapping when biological information and semantic relationship were incorporated into the mapping process. By using the cell line annotation alone (label, synonyms and cross references), 439 out of 624 cell lines were mapped. An additional of 130 cell lines were recovered by integrating biological information and semantic relationship of disease definition. The percent mapping was improved from 70.4 to 91.2% when the biological and semantic content was added on top of the lexical and cross reference mapping criteria.

### Comparison of design patterns

**Fig. 5:**
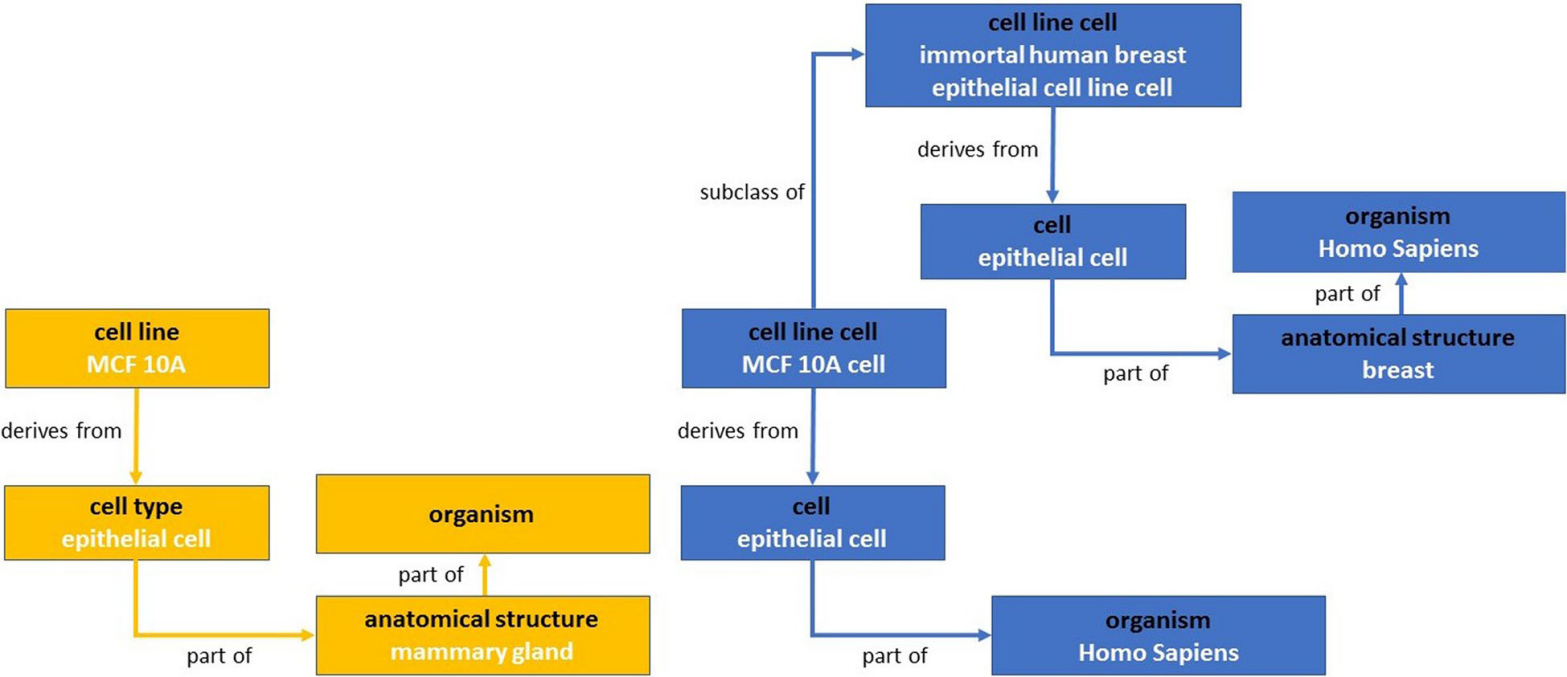
MCF 10A design patterns in EFO and CLO. The EFO cell line design pattern was colored in orange and CLO in *blue*. The *white* text inside each box showed the aligned cell line “MCF 10A” (EFO accession: EFO_0001200; CLO accession: CLO_0007599) with different axiomatized biological information. Additionally, “MCF 10A” cell line cell was defined as “*subclass of*” CLO “immortal human breast epithelial cell line cell”, which added further biological information after hierarchy inference

To illustrate the similarities and differences between CLO and EFO, an example of the aligned cell line, “MCF 10A” is described to facilitate the comparison (Fig. 2). MCF 10A is a non-tumorigenic epithelial cell line derived from mammary gland [19]. In EFO, MCF 10A (EFO_0001200) is directly classified under ‘cell line’ while the CLO ‘MCF 10A cell’ (CLO_0007599) is defined as ‘cell line cell’, and their design patterns are described in Fig. 5. In addition, the CLO cell line class is also a sub-class of ‘immortal human breast epithelial cell line cell’. From the parent term, CLO’s MCF 10A cell inherits the information of human breast epithelial cell. The CLO also includes a more detailed hierarchical definition between ‘cell line cell’ and ‘immortal human breast epithelial cell line cell’ but missing the specific organism part of mammary gland which can be drawn from EFO. Integration of the information from EFO and CLO will enrich the knowledge of the cell line and support better classification.

## Discussion

We have developed a mapping process that combines third-party cross referencing, lexical and biological content comparison, and semantic relation matching in order to compare, align and synchronize immortalized permanent cell lines available in EFO and CLO. In this study, 92.6% (1218 out of 1315) EFO native immortalized permanent cell lines were either aligned or added to CLO. The complementary information integrated from EFO and CLO provided a more comprehensive coverage of immortalized permanent cell lines and their related biological information. In addition, the imported CLO immortalized permanent cell lines defined as the biologically individual cell can help EFO to better represent and model single-cell experiments.

Manual curation of the mapping between ontologies is time consuming, as illustrated here in cell line mapping between EFO and CLO. Our study also proposed an approach to discover new EFO-CLO cell line mapping exploiting the confidence score (C-score) that integrated cell line lexical and semantic information to aid the process of building semi-automated mapping pipeline. Thus, our study illustrated the advantage of utilizing biological content stored as semantic relations in ontology over just lexical comparison for cell line mapping. Using the EFO-Cellosaurus-CLO cross referenced cell lines as the gold standard. In this study, 91.2% of the EFO-CLO cell lines were mapped and validated. Our pipeline that integrated cell line-related biological information and its semantic relations showed improved mapping performance as to lexical matching only. Even though the design of this pipeline is specific for the cell line mapping from EFO to CLO, the usage of biological content and semantic relations defined in the ontologies could be generalized. For example, such information can be applied to probabilistic mapping tools using Bayesian network [20, 21] and Markov network [22] based approaches.

In addition, biological information of a cell line can be examined by their ontology relations to improve the accuracy of the mapping. For example, cell line ‘SW684’ is defined in both EFO and CLO. In EFO, this cell line (EFO_0002369) is derived from ‘connective tissue’. The same cell line in CLO (CLO_0009198) is derived from some ‘fibroblast’. In Cell Ontology (CL) [23], the term ‘fibroblast’ (CL_0000057) is a sub-class of CL term ‘connective tissue cell’ (CL_0002320). Therefore, these two EFO and CLO cell lines can be matched by axiomatization alignment through the biological information provided by CL. The use of ontology in optimal hierarchy identification can further support better cell line mapping performance.

Cell line naming in different laboratories remains an issue of inconsistency, and can lead to repeated usage of cell line names. In our study, we identified two occurrences of the cell line name “H9”. The CLO “H9” cell line is an immortalized human T-cell lymphoma cell line deposited with American Type Culture Collection (ATCC) [24]. The EFO “H9” cell line is a human embryonic stem cell derived from human blastocysts registered in NIH Human Embryonic Stem Cell Registry [25]. Due to the lack of cell line nomenclature standardization or label-usage control by centralized authorities, cell lines derived under different experimental conditions from different organizations at different time points may share the same textual label. Such duplicated cell line labels could lead to confusion when modeling and reporting experimental results. The ambiguity of cell lines should be addressed to identify a differentia to be coded onto the ontology, or by a naming authority of consensus [14, 26].

Another issue throughout the mapping of EFO and CLO cell lines was the imprecise disease definition of a cell line from different resources. This will continue to be a major challenge as modeling of diseases is very difficult due to the dynamic of definition establishment that vary in different clinical communities and expertise. Though much needed, establishing a framework that consolidates the different aspects of disease semantics to be modeled with a common disease ontology is out of the scope of this study. Attempts to find a common ground that consolidates multiple disease vocabularies have been initiated in various projects such as the Monarch Initiative Disease Ontology [21], and the EBI Ontology-cross-Ontology cross-reference mapping service (https://www.ebi.ac.uk/spot/oxo/). Establishing a framework for common disease ontology remains a practice requiring community agreement at large.

## Conclusions

In this study, a mapping process was developed order to compare, align and synchronize immortalized permanent cell lines available in EFO and CLO. The mapping pipeline combined third-party cross referencing, lexical and biological content comparison, and semantic relation matching in. A total of 92.6% EFO native immortalized permanent cell lines were either aligned or added to CLO, and the complementary information was merged. The imported CLO immortalized permanent cell lines defined as the biologically individual cell can help EFO to better represent and model single-cell dynamic under varied experimental conditions.

## Additional files

Additional file 1: Table S1. EFO cell lines drawn from external sources. In the initial step of the EFO-CLO comparison and alignment process, there are 428 and 20 EFO cell lines which were imported from Cell Line Ontology and 20 in BRENDA Tissue and Enzyme Source Ontology respectively. These 448 EFO cell lines were excluded from the entire mapping process. File is stored in Microsoft Excel spreadsheet (xlsx) format. (XLSX 47 kb)

Additional file 2: Table S2. Final EFO-CLO alignment result. The 874 EFO-CLO mapped cell lines aligned and merged into CLO (Tab. 1 in the excel file) and 344 EFO unique immortalized permanent cell lines added to CLO (Tab. 2 in the excel file). File is stored in Microsoft Excel spreadsheet (xlsx) format. (XLSX 54 kb)

## Abbreviations

ATCC: American Type Culture Collection
BTO: BRENDA Tissue and Enzyme Source Ontology
CL: Cell Ontology
CLO: Cell Line Ontology
EBI: European Bioinformatics Institute
EFO: Experimental Factor Ontology
EMBL: European Molecular Biology Laboratory
OBO: Open Biomedical Ontologies
OLS: Ontology Lookup Service
PURL: Permanent URLs
